# Changes in insomnia symptoms among compulsory education students in China after the “Double Reduction” policy: a two-wave longitudinal study

**DOI:** 10.1186/s12888-024-06414-7

**Published:** 2024-12-23

**Authors:** Yifan Zhang, Haoxian Ye, Meijiao Huang, Min Li, Huolian Li, Xiangting Zhang, Junxu Lin, Hao Liu, Hao Wu, Dongfang Wang, Fang Fan

**Affiliations:** School of Psychology, Centre for Studies of Psychological Applications, Guangdong Key Laboratory of Mental Health and Cognitive Science, Ministry of Education Key Laboratory of Brain Cognition and Educational Science, Guangdong Emergency Response Technology Research Center for Psychological Assistance in Emergencies, South China Normal University, Guangzhou, China

**Keywords:** Insomnia, Double Reduction, Stress, Adolescents, Sex difference

## Abstract

**Objective:**

In July 2021, the “Double Reduction” policy was introduced in China, aiming to alleviate the burden of excessive homework and off-campus tutoring for students in the compulsory education stage. The purpose of this study is to explore the changes in students’ insomnia symptoms and related factors after the policy implementation. Meanwhile, sex differences were further examined.

**Methods:**

The baseline survey (T1) began in April 2021 (pre-policy), with a follow-up (T2) conducted in December 2021 (post-policy). A total of 28,398 students completed both T1 and T2 surveys and were included in this study. Insomnia symptoms were measured at both T1 and T2 using three questions from the Youth Self-Report Insomnia Scale. Demographics and anxiety and depressive symptoms were collected at T1, and policy-related indicators were assessed at T2.

**Results:**

The prevalence of insomnia symptoms showed a slight decrease after the “Double Reduction” policy (9.9% vs. 9.2%). After controlling for demographics and anxiety and depressive symptoms, reduced homework (OR: 0.75 [0.65–0.86]), more family time (OR: 0.50 [0.44–0.57]), and reduced academic pressure (OR: 0.77 [0.71–0.83]) post-policy were related to a lower risk of new-onset insomnia symptoms. Additionally, more family time (OR: 0.59 [0.49–0.72]) and reduced academic pressure (OR:0.70 [0.56–0.86]) were factors against persistent insomnia symptoms. There were no significant sex differences in the associations between policy-related indicators and insomnia symptoms.

**Conclusions:**

The “Double Reduction” policy has somewhat improved the students’ insomnia symptoms. Extending family time, and alleviating homework and academic burden are considered measures for maintaining sleep health in students.

**Supplementary Information:**

The online version contains supplementary material available at 10.1186/s12888-024-06414-7.

## Introduction

In China, school-age children and adolescents must receive nine years of free compulsory education, including six years of primary school and three years of middle school. Compulsory education aims to impart basic cultural knowledge to students and to cultivate their comprehensive quality. However, with the rapid social development in recent decades, Chinese students are facing an educational rat race and their academic pressure has significantly increased [1]. In July 2021, the Chinese government proposed a policy aimed at reducing the burden of homework and off-campus tutoring for students in the compulsory education stage, known as the “Double Reduction” policy [2]. This policy outlined 30 specific guidelines, such as comprehensively reducing the total amount of homework, encouraging students to engage in diverse extracurricular activities and more physical exercise, and promoting parent-child interaction, and was officially implemented in September 2021 [2]. Although some studies have confirmed the effectiveness of the policy [3–5], evidence regarding its impact on health issues in students remains insufficient, with insomnia being one of them. Insomnia is a common complaint among adolescents, characterized by difficulty falling asleep, difficulty maintaining sleep, and/or early morning awakening [6]. It is crucial to identify and address insomnia symptoms timely, as it can affect adolescents’ academic engagement and social interactions [7, 8]. In severe cases, it may even lead to the development of mental disorders and suicide [9, 10]. Previous research has identified some factors influencing insomnia among adolescents, e.g., sex, physical activity, emotional problems, and academic pressure [11–14]. However, to our knowledge, most studies within the context of the “Double Reduction” policy have focused on students’ emotional health [3, 15], with no studies examining changes in insomnia symptoms and their associated factors.

Academic pressure, an important factor contributing to school maladaptation and psychosocial issues among adolescents [1], is a key target that the policy aims to intervene. Excessive homework, high parental expectations, and an overemphasis on academic achievement motivation form the long-standing academic pressure experienced by adolescents [1, 16, 17]. Moreover, many people even sacrifice their leisure time or abandon hobbies to prioritize schoolwork [18], particularly within the Confucian cultural background in China, which emphasizes diligence and endurance [19]. Existing evidence provided support for understanding the relationship between academic pressure and insomnia symptoms. Theoretically, exposure to stressful events (e.g., academic pressure) may impair normal sleep functioning [20] and lead to insomnia when individuals’ stress response exceed a threshold [21]. Empirical research has confirmed this connection, showing that exposure to stressors is a predictor for insomnia onset and the likelihood of developing insomnia increases with prolonged stress duration [22]. A systematic review of 34 studies also indicated a close association of academic stress with poor sleep quality and insomnia in adolescents [23]. In addition to academic pressure, aspects of daily life such as exercise, extracurricular activities, and interactions with family are also indicators of the policy interest. Previous research has shown that lifestyle factors are contributors to insomnia [12, 24]. For instance, a large-scale population study indicated that low levels of physical activity and living apart from parents were associated with more insomnia symptoms among Chinese adolescents [12]. Nevertheless, previous studies have predominantly used mean values or prevalence rates to depict sleep problems in specific populations at a particular time point, ignoring the heterogeneity in the change of sleep [12, 23, 24]. In other words, assessments based on a single time point may only capture sleep status at that moment, overlooking the fact that some individuals experience chronic and persistent sleep problems, while others may show remission. This limitation hindered the accurate identification of the diverse patterns of changes in adolescent sleep problems over time, particularly after their experience with the “Double Reduction” policy. To our knowledge, there is still a lack of data quantifying the impact of academic pressure and related daily situations changes post-policy on the changes in insomnia symptoms.

Furthermore, the link between changes in daily situations and insomnia may vary between individuals of different sexes [20]. Illustratively, females are thought to exhibit greater sleep reactivity to stress changes than males [20, 25]. A recent systematic review highlighted sex differences in sleep, noting that females are generally more susceptible to sleep disturbances [26]. Theoretically, physiological differences between males and females become more pronounced after puberty [27]; one such difference is that females typically show higher levels of cortisol arousal in response to stress, which may predispose them to insomnia [28]. Additionally, a meta-analysis of 19 randomized controlled trials found that altered physical activity levels can improve insomnia symptoms and that females may benefit more from physical exercise interventions [29]. These studies suggested that sex differences may also exist in the extent to which insomnia symptoms are influenced by stress and daily situation changes brought about by the “Double Reduction” policy. To our knowledge, no studies have yet examined sex differences in the association between the policy and students’ insomnia symptoms. Identifying these differences could help educational authorities assess the policy’s impacts more accurately and provide a basis for refining intervention measures.

The current study aims to investigate the changes in insomnia symptoms among Chinese adolescents and their related factors after the “Double Reduction” policy implementation by conducting a two-wave survey with a 7-month interval. To better capture the policy’s impact on academic pressure and daily routine, this study used self-made questions to assess individuals’ perceptions of changes in their academic pressure and daily routines following the policy implementation. Three specific objectives are as follows: (1) To describe the prevalence and change patterns of insomnia symptoms among Chinese adolescents before and after the implementation of the “Double Reduction” policy; (2) To explore the associations of academic pressure and related daily situation changes with the change patterns of insomnia symptoms; (3) To examine whether there are sex differences in the associations between the policy-induced changes and change patterns of insomnia symptoms.

## Methods

### Procedure and participants

This study was conducted in 152 primary and middle schools in a district of Shenzhen City, Guangdong Province. We employed a two-wave longitudinal design with a 7-month interval. As described in previous research by our team [3], the baseline survey (Time 1, T1) was conducted from April 21st to May 12th, 2021 (i.e., four months before the implementation of the “Double Reduction” Policy). The follow-up survey (Time 2, T2) was conducted between December 17th and 26th, 2021 (i.e., three months after the implementation of the “Double Reduction” Policy). Both waves of surveys were conducted under the unified organization of the regional education bureau. Specifically, the education bureau distributed an online survey and invitation letter to target schools. Then, all schools forwarded the questionnaire and letter to students’ parents within the stipulated timeframe, and students could use their parents’ mobile phones to participate in the survey after school or on weekends. Before completing the questionnaire, students and their guardians were required to read an informed consent form. They were informed of the confidentiality measures of this study and that they could freely terminate answering without any penalty. It was estimated that the total number of students in these schools was approximately 300,000. The present study obtained approval from the Ethics Committee of South China Normal University (SCNU-PSY-2021-094) and the research procedures strictly adhered to the principles of the Helsinki Declaration.

Students in the compulsory education stage (grades 1–6 for primary school and grades 7–9 for middle school) were the target population of this study. However, those in grades 1–4 were not included in the study due to their young age and potentially limited comprehension of the questionnaire. The 9th graders were also excluded as they were about to graduate and could not participate in follow-up surveys. Altogether, 110,211 and 94,624 students were recruited in the T1 and T2 surveys, respectively. Three exclusion criteria were employed to ensure data quality: (1) abnormal questionnaire completion times (e.g., completion times less than the total number of items * 2 s); (2) extreme response biases (such as selecting the same option for all items on the Likert scale); and (3) self-reported having a current or history of mental illness. After data screening, 101,976 (92.5%) and 87,449 (92.4%) eligible subjects remained for T1 and T2 surveys, respectively. Among these eligible participants, 28,398 students provided complete data across both waves of surveys and they were included in the subsequent analysis.

### Measures

#### Insomnia symptoms

Three items from the Youth Self-Report Insomnia Scale (YSIS) [30] were used to assess insomnia symptoms. These items inquire about the frequency of core symptoms of insomnia (i.e., difficulty initiating sleep, difficulty maintaining sleep, and early morning awakening) experienced by the participants in the past month. Each item is scored on a five-point scale ranging from 0 (never) to 4 (6–7 times per week). Referring to prior research [31, 32], participants who chose a response option of 3 or above (≥ 3 times per week) on any item were considered to have insomnia symptoms. This measurement has been demonstrated to possess good reliability and validity in adolescent populations [30, 33]. Cronbach’s α for these three items was 0.78 in both surveys of this study.

#### “Double Reduction” policy-related indicators

Focusing on the specific regulations proposed by the “Double Reduction” policy [2], the research team designed five items to assess the students’ daily situations after the implementation of the “Double Reduction” policy, including five aspects of “reduced homework”, “more extracurricular activities”, “increased physical activity”, “more family time”, and “reduced academic pressure” [3]. For the homework and academic pressure indicators, participants’ response options included “1 = significantly decreased”, “2 = slightly decreased”, and “3 = no changes or increased”. For the other three indicators, the response options included “1 = significantly increased”, “2 = slightly increased”, and “3 = no change or decreased”. In this study, we recoded these five indicators into binary variables, with the original options 1 and 2 merged into 1 (Yes), and the original option 3 was recoded as 2 (No). In the T2 survey, Cronbach’s α for the five items was 0.82.

#### Anxiety and depressive symptoms

The Patient Health Questionnaire-4 (PHQ-4) is a brief self-report tool utilized for screening core symptoms of anxiety and depressive symptoms (i.e., being nervous or anxious, being unable to stop or control worrying, decreased interest in activities, and feeling hopeless) in the past two weeks [34]. For each item, participants scored on a scale of 0 (not at all) to 3 (nearly every day), with a total score ranging from 0 to 12. PHQ-4 scores of 6 to 12 can be interpreted as having moderate-to-severe anxiety and depressive symptoms. The questionnaire has been proven to have good psychometric properties [35]. Cronbach’s α for PHQ-4 was 0.86 and 0.88 in the T1 and T2 surveys, respectively.

#### Other covariates

Social-demographical factors included age (continuous), sex (female, male), grade (grade 5 to 8), school types (private school, public school), boarding at school (yes, no), number of siblings (one, more than one), parental marital status (married, separated/divorced/widowed), family income (monthly) (< ¥12,000, ¥12,000-¥30,000, > ¥30,000, unknown), suffering chronic somatic diseases (yes, no), and family history of mental illness (yes, no).

### Statistical analysis

First, descriptive analysis was performed to outline sample characteristics, presenting categorical data as frequencies and percentages, and quantitative data as means and standard deviations. To describe changes in the prevalence of insomnia symptoms, we conducted two types of comparisons. The first was a direct observational comparison of insomnia prevalence between the two cross-sectional survey samples at T1 (*N* = 101,976) and T2 (*N* = 87,449). The second involved using McNemar’s test to compare differences in insomnia prevalence within the longitudinal sample (*N* = 28,398) between T1 and T2. Subsequently, this study classified participants into four groups based on the occurrence of insomnia symptoms in two survey waves: resistance group (no insomnia symptoms were observed in either T1 or T2), remission group (having insomnia symptoms in T1 but none in T2), new-onset group (having insomnia symptoms in T2 but none in T1), and persistence group (suffering insomnia symptoms in both T1 and T2). This classification has been utilized in prior research [36, 37]. Then, the Chi-square test was employed to compare differences in social-demographical characteristics, and “Double Reduction” policy-related indicators across various groups. Multivariate logistic regressions were conducted to examine predictors for the changes in insomnia symptoms. We designated the resistance group as the reference group to explore the protective and risk factors of the new-onset groups. We also compared the remission group and persistence group to examine the influential factors associated with the alleviation of insomnia symptoms. Odds ratios (ORs) and 95% confidence intervals (95% CI) were calculated to quantify the strength of the association between variables. Furthermore, sex-stratified logistic regression was performed to examine sex differences in the associations between policy-related indicators and changes in insomnia symptoms. Interaction terms between sex and policy-related indicators were included in the model simultaneously, and the *p*-values for these interactions were reported. Finally, to further verify the relationship between the policy-related indicators and different change patterns of insomnia symptoms, this study adopted Propensity Score Matching (PSM) based on all demographic characteristics and anxiety and depressive symptoms to perform 1:1 matching for participants in the new-onset and resistance groups, as well as the persistence and remission groups and conducted sensitivity analysis using the matched data. PSM was conducted in R version 4.2.1, and the remaining analyses were performed using IBM SPSS Statistics for Version 25.0. The Sankey diagram was drawn to visualize the change patterns of insomnia symptoms. Considering the large sample size of this study, a two-tailed *p*-value < 0.001 was considered statistically significant.

## Results

### Sample characteristics

As shown in Table 1, the average age of the 28,398 participants was 12.3 (1.6) years. Among them, 13,417 (47.2%) students were females, 14,461 (50.9%) attended private schools, 2,317 (8.2%) boarded at school, 5,638 (19.9%) had no siblings, and 2,203 (7.8%) individuals reported experiencing moderate-to-severe anxiety and depressive symptoms. In addition, the response distribution of the policy-related indicators is presented in Supplementary Fig. 1.

**Table 1:** Sample characteristics (*N* = 28,398)

Variables	*n*	%
Sex (female)	13,417	47.2
Grade [age, years]		
Grade 5 [11.1 ± 0.6]	10,545	37.1
Grade 6 [12.1 ± 0.6]	5,991	21.1
Grade 7 [13.0 ± 0.6]	7,030	24.8
Grade 8 [14.0 ± 0.6]	4,832	17.0
School types (private school)	14,461	50.9
Boarding at School (yes)	2,317	8.2
Number of siblings (one)	5,638	19.9
Parental marital status (poor ^a^)	1,351	4.8
Family income (monthly)		
< ¥ 12,000	12,506	44.0
¥ 12,000- ¥ 30,000	7,423	26.1
> ¥ 30,000	2,342	8.2
Unknown	6,127	21.6
Chronic somatic diseases (yes)	984	3.5
Family history of mental illness (yes)	267	0.9
Anxiety and depressive symptoms (yes)	2,203	7.8
Reduced homework (yes)	22,901	80.6
More extracurricular activities (yes)	20,137	70.9
Increased physical activity (yes)	22,483	79.2
More family time (yes)	18,366	64.7
Reduced academic pressure (yes)	13,129	46.2
Insomnia symptoms at T1 (yes)	2,802	9.9
Insomnia symptoms at T2 (yes)	2,609	9.2

^a^ Poor parental marital status included separated, divorced, and widowed

### Changes in insomnia symptoms after the “Double Reduction” policy

**Fig. 1:**
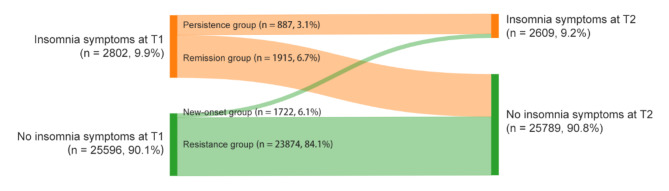
Change patterns of insomnia symptoms

In the two cross-sectional survey samples at T1 (*N* = 101,976) and T2 (*N* = 87,449), the prevalence of insomnia symptoms was 10.6% and 9.6%, respectively. In the longitudinal sample (*N* = 28,398), the prevalence of insomnia symptoms after the “Double Reduction” policy was 9.2%, showing a slight decrease compared to the prevalence of 9.9% before the “Double Reduction” policy *(χ²* = 10.14, *p* < 0.001, Cohen’s φ = 0.019) (see Table 1). Moreover, we further distinguished four patterns of changes in insomnia symptoms from before to after the “Double Reduction” policy (see Fig. 1). Most participants had been in the resistance group (84.1%), and 6.7% were classified in the remission group. The proportions of the new-onset and persistence groups were 6.1% and 3.1%, respectively.

**Fig. 2:**
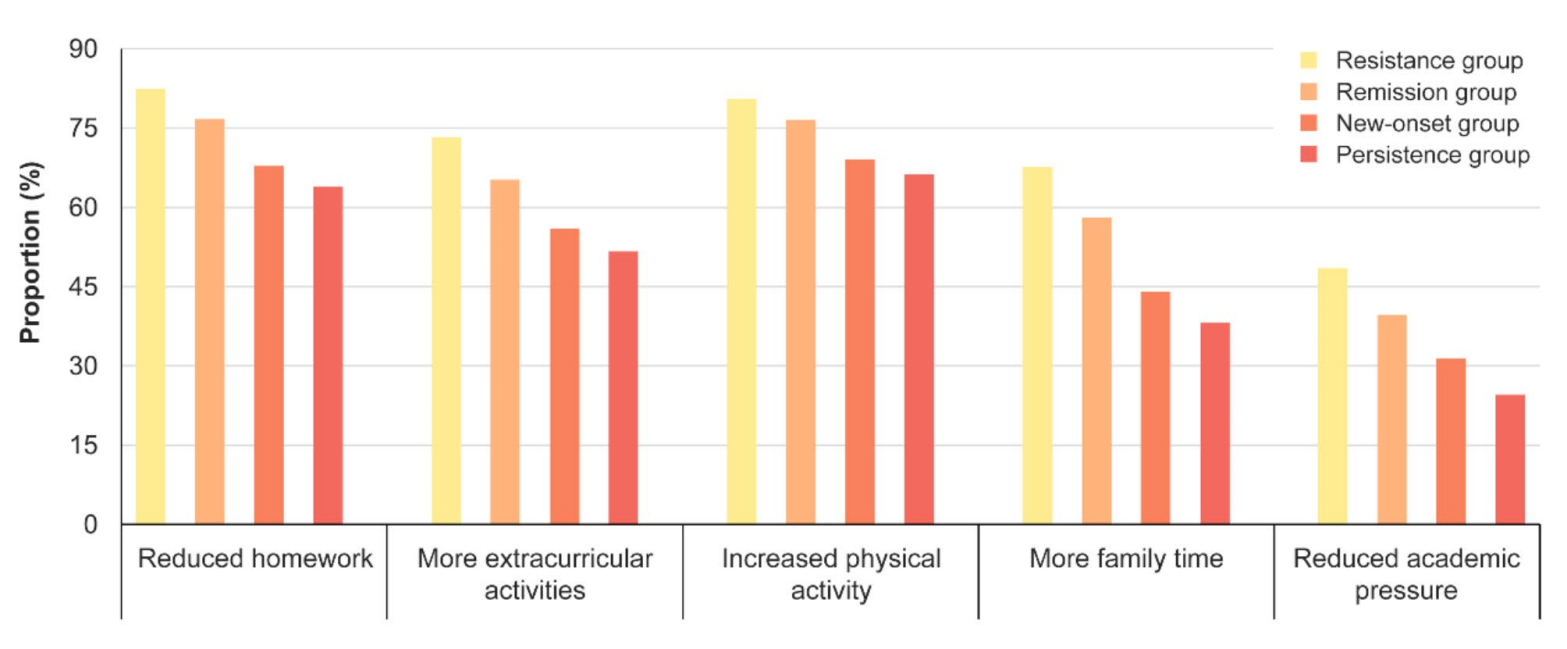
“Double Reduction” policy-related indicators among different groups of insomnia symptoms. Note: Significant differences were observed in all “Double Reduction” policy-related variables among the four groups (all *p* < 0.001)

Table 2 displays differences among the four groups in sociodemographic characteristics. Chi-square test results indicated that individuals who were female, boarded at school, had poor parental marital status, experienced chronic somatic diseases, and reported anxiety and depressive symptoms were more likely to be classified into the group of new-onset or persistence (all *p* < 0.001). Moreover, the comparative results among different groups on the “Double Reduction” policy-related indicators are illustrated in Fig. 2. The resistance group reported the highest proportions of reduced homework”, more extracurricular activities, increased physical activity, more family time, and reduced academic pressure, successively followed by the remission group, new-onset group, and persistence group (all *p* < 0.001).

**Table 2:** Comparisons of demographic characteristics among different groups of insomnia symptoms

Variables	Changes in insomnia symptoms, *n* (%)				χ²	*p*-Value
	Resistance (*n* = 23,874)	Remission (*n* = 1,915)	New-onset (*n* = 1,722)	Persistence (*n* = 887)		
Sex					121.89	< 0.001
Male	12,904 (86.1)	895 (6.0)	834 (5.6)	348 (2.3)		
Female	10,970 (81.8)	1,020 (7.6)	888 (6.6)	539 (4.0)		
Grade [age, years]					76.07	< 0.001
Grade 5 [11.1 ± 0.6]	9,024 (85.6)	638 (6.0)	621 (5.9)	262 (2.5)		
Grade 6 [12.1 ± 0.6]	5,075 (84.7)	430 (7.2)	316 (5.3)	170 (2.8)		
Grade 7 [13.0 ± 0.6]	5,815 (82.7)	482 (6.9)	485 (6.9)	248 (3.5)		
Grade 8 [14.0 ± 0.6]	3,960 (81.9)	365 (7.6)	300 (6.2)	207 (4.3)		
School types					3.45	0.328
Public school	11,659 (83.7)	969 (6.9)	858 (6.2)	448 (3.2)		
Private school	12,215 (84.5)	946 (6.5)	864 (6.0)	439 (3.0)		
Boarding at school					48.09	< 0.001
No	22,031 (84.5)	1,689 (6.5)	1,567 (6.0)	794 (3.0)		
Yes	1,843 (79.5)	226 (9.8)	155 (6.7)	93 (4.0)		
Number of siblings					8.96	0.030
More than one	19,083 (83.9)	1,579 (6.9)	1,372 (6.0)	726 (3.2)		
One	4,791 (85.0)	336 (6.0)	350 (6.2)	161 (2.8)		
Parental marital status					48.47	< 0.001
Married	22,812 (84.3)	1,806 (6.7)	1,622 (6.0)	807 (3.0)		
Separated/divorced/widowed	1,062 (78.6)	109 (8.1)	100 (7.4)	80 (5.9)		
Family income (monthly)					59.46	< 0.001
< ¥ 12,000	10,634 (85.0)	775 (6.2)	750 (6.0)	347 (2.8)		
¥ 12,000- ¥ 30,000	6,256 (84.3)	472 (6.4)	478 (6.4)	217 (2.9)		
> ¥ 30,000	1,972 (84.2)	170 (7.3)	130 (5.5)	70 (3.0)		
Unknown	5,012 (81.8)	498 (8.1)	364 (6.0)	253 (4.1)		
Chronic somatic diseases					78.50	< 0.001
No	23,126 (84.4)	1,812 (6.6)	1,658 (6.0)	818 (3.0)		
Yes	748 (76.0)	103 (10.5)	64 (6.5)	69 (7.0)		
Family history of mental illness					36.60	< 0.001
No	23,682 (84.2)	1,875 (6.7)	1,700 (6.0)	874 (3.1)		
Yes	192 (71.9)	40 (15.0)	22 (8.2)	13 (4.9)		
Anxiety and depressive symptoms					3425.36	< 0.001
No	22,869 (87.3)	1,335 (5.1)	1,488 (5.7)	503 (1.9)		
Yes	1,005 (45.6)	580 (26.3)	234 (10.6)	384 (17.5)		

### Factors associated with change patterns of insomnia symptoms

As shown in Table 3, individuals with anxiety and depressive symptoms were more likely to have newly onset insomnia symptoms (vs. resistance group, OR [95% CI]: 2.91 [2.49–3.41]) and persistent insomnia symptoms (vs. remission group, OR [95% CI]: 1.52 [1.28–1.81]). As for the “Double Reduction” policy-related indicators, the likelihood of developing new insomnia symptoms decreased if the participants reported reduced homework (vs. resistance group, OR [95% CI]: 0.75 [0.65–0.86]). Students who reported more family time were significantly associated with a reduced risk of new-onset insomnia symptoms (vs. resistance group, OR [95% CI]: 0.50 [0.44–0.57]) as well as persistent insomnia symptoms (vs. remission group, OR [95% CI]: 0.59 [0.49–0.72]). Additionally, those with reduced academic pressure had a lower risk of being in the new-onset group (vs. resistance group, OR [95% CI]: 0.77 [0.71–0.83]) and the persistence group (vs. remission group, OR [95% CI]: 0.70 [0.56–0.86]).

**Table 3:** Risk and protective factors of change in insomnia symptoms

	OR (95% CI)	
	New-onset vs. Resistance	Persistence vs. Remission
**Demographic characteristics**		
Sex (male as Ref.)	1.09 (0.99–1.21)	1.11 (0.93–1.32)
Grade (grade 8 as Ref.)		
Grade 5	1.25 (1.05–1.47)^**^	1.00 (0.78–1.27)
Grade 6	1.06 (0.90–1.26)	0.81 (0.63–1.06)
Grade 7	1.23 (1.06–1.44)^**^	0.98 (0.77–1.24)
Private school (public school as Ref.)	0.98 (0.88–1.09)	1.04 (0.87–1.25)
Boarding at School (no as Ref.)	1.16 (0.96–1.40)	0.78 (0.58–1.04)
No sibling (more than one sibling as Ref.)	0.98 (0.86–1.11)	0.98 (0.78–1.22)
Poor parental marital status (married as Ref.)	1.20 (0.97–1.49)	1.54 (1.11–2.12)^**^
Family income (monthly) (< ¥ 12,000 as Ref.)		
¥ 12,000- ¥ 30,000	1.07 (0.94–1.21)	1.08 (0.87–1.34)
> ¥ 30,000	0.90 (0.73–1.09)	0.97 (0.70–1.34)
Unknown	0.95 (0.83–1.08)	1.14 (0.93–1.40)
Chronic somatic diseases (no as Ref.)	1.03 (0.79–1.34)	1.45 (1.05–2.01)^*^
Family history of mental illness (no as Ref.)	1.26 (0.80–1.99)	0.59 (0.31–1.14)
Anxiety and depressive symptoms (no as Ref.)	**2.91 (2.49–3.41)** ^***^	**1.52 (1.28–1.81)** ^***^
**“Double Reduction” policy-related variables**		
Reduced homework (no as Ref.)	**0.75 (0.65–0.86)** ^***^	0.83 (0.67–1.02)
More extracurricular activities (no as Ref.)	0.88 (0.76–1.01)	1.03 (0.82–1.29)
Increased physical activity (no as Ref.)	1.04 (0.91–1.19)	0.89 (0.72–1.10)
More family time (no as Ref.)	**0.50 (0.44–0.57)** ^***^	**0.59 (0.49–0.72)** ^***^
Reduced academic pressure (no as Ref.)	**0.77 (0.71–0.83)** ^***^	**0.70 (0.56–0.86)** ^***^

Note: ^*^p < 0.05, ^**^p < 0.01, ^***^p < 0.001. OR, odds ratio; 95%CI, 95% confidence interval; Ref, reference

### **Sex differences in relationships between policy-related indicators and change patterns of insomnia symptoms**

As shown in Table 4, after controlling for demographic variables and anxiety and depressive symptoms, males who reported reduced homework were significantly associated with a lower risk of new-onset insomnia symptoms (vs. resistance group, OR [95% CI]: 0.70 [0.58–0.86]), while this association was weaker among females (vs. resistance group, OR [95% CI]: 0.80 [0.66–0.95]). The associations of more family time with new-onset insomnia symptoms (vs. resistance group, OR [95% CI] for males: 0.47 [0.39–0.56], OR [95% CI] for females: 0.54 [0.45–0.64]) and persistent insomnia symptoms (vs. remission group, OR [95% CI] for males: 0.55 [0.40–0.75], OR [95% CI] for females: 0.62 [0.48–0.81]) were significant in both males and females. Moreover, reduced academic pressure was a protective factor against the development of insomnia symptoms in females (new-onset vs. resistance, OR [95% CI]: 0.68 [0.57–0.82]) rather than males. Despite the difference in effect sizes, the interaction effects analysis showed that all interaction terms between sex and each policy-related indicator were not significant (*p*-values for interaction ranged from 0.071 to 0.948).

**Table 4:** Sex-stratified logistic regression analysis for policy-related indicators and changes in insomnia symptoms

	New-onset vs. Resistance, OR (95% CI) ^a^		*p* for interaction	Persistence vs. Remission, OR (95% CI) ^a^		*p* for interaction
	Male	Female		Male	Female	
Reduced homework			0.370			0.756
No	1.00 (reference)	1.00 (reference)		1.00 (reference)	1.00 (reference)	
Yes	**0.70 (0.58–0.86)** ^***^	0.80 (0.66–0.95)^*^		0.81 (0.57–1.14)	0.86 (0.66–1.13)	
More extracurricular activities			0.948			0.862
No	1.00 (reference)	1.00 (reference)		1.00 (reference)	1.00 (reference)	
Yes	0.88 (0.71–1.07)	0.87 (0.72–1.05)		1.04 (0.72–1.49)	1.00 (0.75–1.33)	
Increased physical activity			0.397			0.853
No	1.00 (reference)	1.00 (reference)		1.00 (reference)	1.00 (reference)	
Yes	0.98 (0.80–1.19)	1.12 (0.93–1.34)		0.91 (0.64–1.29)	0.88 (0.67–1.16)	
More family time			0.284			0.538
No	1.00 (reference)	1.00 (reference)		1.00 (reference)	1.00 (reference)	
Yes	**0.47 (0.39–0.56)** ^***^	**0.54 (0.45–0.64)** ^***^		**0.55 (0.40–0.75)** ^***^	**0.62 (0.48–0.81)** ^***^	
Reduced academic pressure			0.071			0.851
No	1.00 (reference)	1.00 (reference)		1.00 (reference)	1.00 (reference)	
Yes	0.86 (0.72–1.03)	**0.68 (0.57–0.82)** ^***^		0.70 (0.51–0.97)^*^	0.69 (0.52–0.92)^*^	

**Abbreviations:** OR, odds ratio; 95% CI, 95% confidence interval

^a^ Adjusted for grade, school types, boarding at school, number of siblings, parental marital status, family income, chronic somatic diseases, family history of mental illness, and anxiety and depressive symptoms

### Sensitivity analysis

Supplementary Tables 1 and Supplementary Table 2 show the sample characteristics based on PSM. There were 1,722 participants each in the new-onset and resistance groups, and 887 participants each in the persistence and remission groups. There were no significant differences in any demographic characteristics and anxiety and depressive symptoms between the matched groups. Regarding the relationship between the “Double Reduction” policy-related indicators and insomnia symptom change patterns and their sex differences, the sensitivity analysis revealed consistent results (see Supplementary Tables 3 and Supplementary Table 4).

## Discussion

The current study examined the changes in insomnia symptoms among compulsory education students in China after the “Double Reduction” policy was implemented using large-scale population-based data. We further investigated the association of the policy’s impact on student’s academic pressure and daily life dynamics and the changes in insomnia symptoms, as well as the sex differences in these associations.

In this study, the prevalence of insomnia symptoms showed a slight decrease after the implementation of the “Double Reduction” policy, both in the two cross-sectional survey samples (10.6% vs. 9.6%) and in the longitudinal sample (9.9% vs. 9.2%). This finding suggested that the policy may have positive influences on sleep health in adolescents, echoing previous literature that increased homework time and academic burden would correspondingly shorten sleep duration and impair sleep quality [14, 17]. Several inferences may help understand this trend. Firstly, the follow-up survey for this study was conducted three months after the official implementation of the policy, during which the initiative was in full swing. Schools, communities, and parents, driven by the government’s active promotion, made concerted efforts to ensure the enforcement of the policy recommendations. As a result, students’ academic pressure might have been temporarily alleviated due to this social atmosphere, leading to an improvement in their sleep conditions. Secondly, in the context of “Double Reduction”, the allocation of students’ daily activities may undergo adjustments. For instance, previous studies have reported that after the implementation of the policy, students’ exercise frequency, sleep duration, and time spent communicating with family increased compared to before, while time spent on homework and using electronic devices significantly decreased [3, 4, 38, 39]. These adaptive changes could also be reasons for the reduction in insomnia symptoms [40]. However, the evidence supporting these inferences remains limited and requires further investigation. Moreover, it is needed to acknowledge that the effect size of the decrease in insomnia symptoms among the longitudinal sample is small (*χ²* = 10.14, Cohen’s φ = 0.019), and the stability of the observed trend requires more data for verification. As adolescents age, they may experience more sleep problems [41], which could potentially influence the effectiveness of the “Double Reduction” policy.

This study distinguished four change patterns of insomnia symptoms, with the majority of individuals exhibiting no or mild insomnia symptoms in both surveys (84.1%). It is consistent with previous research that approximately over half of adolescents maintain good sleep function over time [42–44]. Research by Fernandez-Mendoza et al. also suggested that nearly 50% of individuals reported no insomnia symptoms from childhood to young adulthood [45]. Moreover, in our study, the proportions of students with newly developed and persistent insomnia symptoms were 6.1% and 3.1%, respectively. A potential explanation for those who experienced new-onset or persistent insomnia symptoms could be related to adolescent development. The age range of participants was between 10 and 15 years old, indicating that they might be approaching or going through puberty. Hence, their insomnia symptoms might have continued or worsened during the follow-up period due to physiological transitions associated with adolescence [46].

As expected, even under the “Double Reduction” context, anxiety and depressive symptoms remained a primary factor related to insomnia symptoms. This association has been confirmed in previous studies [47, 48]. On one hand, insomnia is often a somatic manifestation of emotional problems characterized by anxiety and depression [49]. On the other hand, adolescents reporting anxiety and depressive symptoms may have a higher susceptibility to stress and adversity, resulting in an increased likelihood of sleep disturbance emerging [50]. In addition, our analysis of policy-related measures revealed that more family time is the most closely associated index with insomnia symptoms, both in terms of symptom alleviation and prevention. Numerous studies have demonstrated the role of the family in addressing sleep problems. More family time may imply better family functioning, a more harmonious family atmosphere, emotional expression, and parental support [51], all of which play an important protective role in preventing insomnia [44, 52]. Moreover, increased parental support may also help adolescents cope positively with academic stress [53]. The above findings further underscore the importance of parental companionship in adolescent sleep health, especially within the background of escalating social competition.

Our results indicated that reduced homework was associated with a lower odd of new-onset insomnia symptoms in total samples. The negative association between reduced homework and insomnia symptoms is easily understandable. Prior research has shown that less homework/studying time among adolescents was related to increased sleep duration and decreased negative emotions [17], which may indirectly improve insomnia issues. Those with reduced homework are more likely to allocate the time previously spent on homework to engage in physical activity and sleep [54], implying a healthier lifestyle, which in turn plays a protective role in sleep health [12]. In this study, the positive relationship between reduced sense of academic pressure and decreased risk of insomnia symptoms was observed in total samples. This finding aligns with the stress-diathesis model of insomnia, where insomnia symptoms are closely associated with stressors [20]. There may be underlying physiological mechanisms behind this phenomenon. For example, chronic stress can lead to sustained activation of the hypothalamic-pituitary-adrenal axis [55]. This process is accompanied by heightened somatic arousal and increased cortisol levels over a 24-hour period [55], which is an overarching pathophysiological condition in insomnia patients [56].

It is important to note that, although the strength and significance levels of the associations of policy-related indicators with insomnia symptoms differed in the sex-stratified analysis, the interactions between sex and each of these indicators were not statistically significant. Previous research suggested that females tend to exhibit greater sleep reactivity when facing stress or changes in life circumstances [20, 27]. However, this study did not observe a similar phenomenon. This may be because the “Double Reduction” policy targets stress factors commonly faced by adolescents, such as excessive academic workload and limited family interaction, which may have a similar positive impact across sexes. Additionally, the policy was implemented uniformly and consistently across schools, resulting in similar perceptions of change among students, potentially diminishing sex differences.

The strengths of the current study included the use of a large-scale sample and a two-wave follow-up design, which allowed us to compare changes in insomnia symptoms among compulsory education students in China before and after the implementation of the “Double Reduction” policy. However, several limitations should be acknowledged. First, insomnia symptoms were measured by self-report questionnaires rather than clinical interviews, which inevitably introduced recall bias. Moreover, this study defined insomnia symptoms based on the DSM-V criteria for insomnia disorder [6]. Although this definition and the corresponding assessment of insomnia symptoms have been frequently utilized in previous population-based studies [32, 33], there remains a lack of rigorous clinical data to substantiate its clinical relevance. Second, the “Double Reduction” policy-related indicators were treated as binary variables, which may have led to some loss of information provided by the data and limited the explanatory power of the policy’s impact. Additionally, the interval between the two waves of surveys was only seven months, which may not be sufficient to capture the long-term effects of the policy. Ongoing follow-up surveys are needed to examine whether the benefits are stable over the long term. Third, the present study was conducted in the real world, so it must be acknowledged that changes in insomnia symptoms could still be influenced by other factors (e.g., natural physiological development of adolescents, life events, and COVID-19-related factors), even though the implementation of the “Double Reduction” policy was the most significant event during the survey period and we controlled for baseline anxiety and depressive symptoms. Meanwhile, the T1 and T2 surveys were conducted during the spring and autumn semesters, respectively, which may also represent a limitation, as seasonal differences could potentially impact the results. Fourth, although this study included school type as a school-level factor in the model, it did not specifically analyze differences between individual schools. The varying policy response measures across schools may also potentially influence the policy’s effectiveness.

## Conclusions

A declining trend in insomnia symptoms among students in the compulsory education stage in China was observed after the “Double Reduction” Policy. Reduced homework, increased family time, and decreased academic pressure brought about by the policy are associated with a lower risk of insomnia symptoms. Moreover, the policy-induced changes show no significant sex differences in their association with insomnia symptoms. Designing insomnia interventions aiming at promptly addressing emotional problems, extending the time for interaction with family members, and alleviating homework loads and academic pressures is important for maintaining and promoting students’ sleep health.

## Electronic supplementary material

Below is the link to the electronic supplementary material.

Supplementary Material 1

## Data Availability

The data used in the current study is available from the corresponding author (FF) upon reasonable request.
