# Supplementary material for: Changes in insomnia symptoms among compulsory education students in China after the “Double Reduction” policy: a two-wave longitudinal study
Source: BMC Psychiatry. 2024 Dec 23;24:945. doi: 10.1186/s12888-024-06414-7 (PMC11667910; doi:10.1186/s12888-024-06414-7)
Supplement: Supplementary file 1 — Supplementary Material 1 [file 12888_2024_6414_MOESM1_ESM.docx]

**
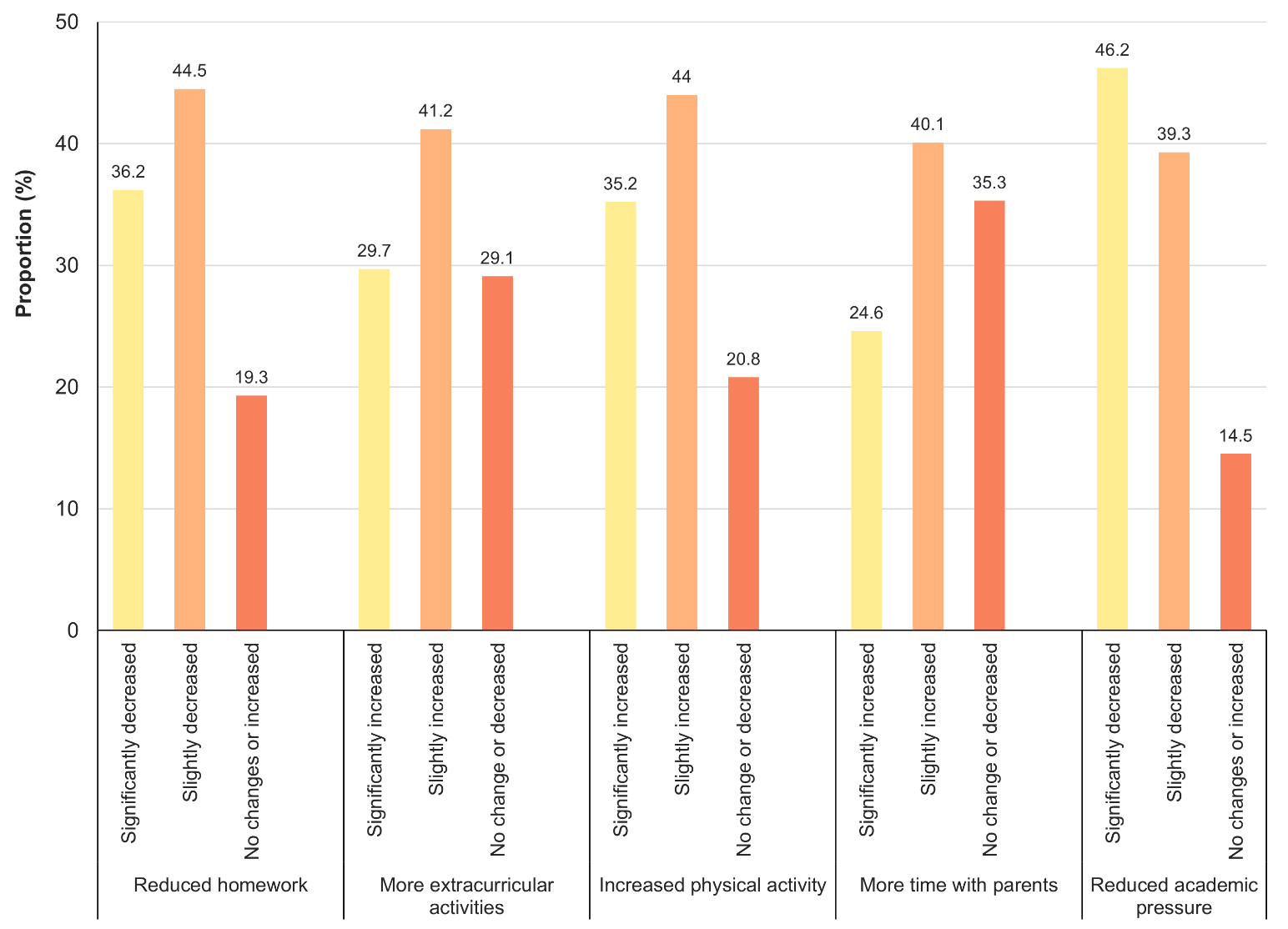
**

**Supplementary Figure 1.** The response distribution of the “Double Reduction” policy-related indicators.

**Supplementary Table 1.** Comparisons of demographic characteristics between resistance and new-onset groups (matched).

| Variables | Changes in insomnia symptoms, n (%) | |  | *χ²* | *p*-Value |
| --- | --- | --- | --- | --- | --- |
|  | Resistance  (n = 1,722) | New-onset  (n = 1,722) |  |  |  |
| Sex |  |  |  | 0.01 | 0.973 |
| Male | 833 (50.0) | 834 (50.0) |  |  |  |
| Female | 889 (50.0) | 888 (50.0) |  |  |  |
| Grade [age, years] |  |  |  | 0.25 | 0.970 |
| Grade 5 [11.1 ± 0.6] | 611 (49.6) | 621 (50.4) |  |  |  |
| Grade 6 [12.1 ± 0.6] | 316 (50.0) | 316 (50.0) |  |  |  |
| Grade 7 [13.0 ± 0.6] | 485 (50.0) | 485 (50.0) |  |  |  |
| Grade 8 [14.0 ± 0.6] | 310 (50.8) | 300 (49.2) |  |  |  |
| School types |  |  |  | 0.12 | 0.733 |
| Public school | 868 (50.3) | 858 (49.7) |  |  |  |
| Private school | 854 (49.7) | 864 (50.3) |  |  |  |
| Boarding at School |  |  |  | 0.01 | 0.905 |
| No | 1,565 (50.0) | 1,567 (50.0) |  |  |  |
| Yes | 157 (50.3) | 155 (49.7) |  |  |  |
| Number of siblings |  |  |  | 0.00 | 1.000 |
| More than one | 1,372 (50.0) | 1,372 (50.0) |  |  |  |
| One | 350 (50.0) | 350 (50.0) |  |  |  |
| Parental marital status |  |  |  | 0.81 | 0.368 |
| Married | 1,634 (50.2) | 1,622 (49.8) |  |  |  |
| Separated/divorced/widowed | 88 (46.8) | 100 (53.2) |  |  |  |
| Family income (monthly) |  |  |  | 0.09 | 0.993 |
| < ¥ 12,000 | 750 (50.0) | 750 (50.0) |  |  |  |
| ¥ 12,000- ¥ 30,000 | 484 (50.3) | 478 (49.7) |  |  |  |
| > ¥ 30,000 | 130 (50.0) | 130 (50.0) |  |  |  |
| Unknown | 358 (49.6) | 364 (50.4) |  |  |  |
| Chronic somatic diseases |  |  |  | 0.01 | 0.928 |
| No | 1,657 (50.0) | 1,658 (50.0) |  |  |  |
| Yes | 65 (50.4) | 64 (49.6) |  |  |  |
| Family history of mental illness |  |  |  | 0.19 | 0.659 |
| No | 1,697 (50.0) | 1,700 (50.0) |  |  |  |
| Yes | 25 (53.2) | 22 (46.8) |  |  |  |
| Anxiety and depressive symptoms |  |  |  | 0.00 | 1.000 |
| No | 1,488 (50.0) | 1,488 (50.0) |  |  |  |
| Yes | 234 (50.0) | 234 (50.0) |  |  |  |

**Supplementary Table 2.** Comparisons of demographic characteristics between remission and persistence groups (matched).

| Variables | Changes in insomnia symptoms, n (%) | |  | *χ²* | *p*-Value |
| --- | --- | --- | --- | --- | --- |
|  | Remission  (n = 887) | Persistence  (n = 887) |  |  |  |
| Sex |  |  |  | 0.19 | 0.662 |
| Male | 357 (50.6) | 348 (49.4) |  |  |  |
| Female | 530 (49.6) | 539 (50.4) |  |  |  |
| Grade [age, years] |  |  |  | 0.42 | 0.936 |
| Grade 5 [11.1 ± 0.6] | 253 (49.1) | 262 (50.9) |  |  |  |
| Grade 6 [12.1 ± 0.6] | 171 (50.1) | 170 (49.9) |  |  |  |
| Grade 7 [13.0 ± 0.6] | 259 (51.1) | 248 (48.9) |  |  |  |
| Grade 8 [14.0 ± 0.6] | 204 (49.6) | 207 (50.4) |  |  |  |
| School types |  |  |  | 0.51 | 0.476 |
| Public school | 463 (50.8) | 448 (49.2) |  |  |  |
| Private school | 424 (49.1) | 439 (50.9) |  |  |  |
| Boarding at School |  |  |  | 1.08 | 0.298 |
| No | 807 (50.4) | 794 (49.6) |  |  |  |
| Yes | 80 (46.2) | 93 (53.8) |  |  |  |
| Number of siblings |  |  |  | 0.03 | 0.853 |
| More than one | 729 (50.1) | 726 (49.9) |  |  |  |
| One | 158 (49.5) | 161 (50.5) |  |  |  |
| Parental marital status |  |  |  | 2.78 | 0.095 |
| Married | 826 (50.6) | 807 (49.4) |  |  |  |
| Separated/divorced/widowed | 61 (43.3) | 80 (56.7) |  |  |  |
| Family income (monthly) |  |  |  | 1.39 | 0.709 |
| < ¥ 12,000 | 356 (50.6) | 347 (49.4) |  |  |  |
| ¥ 12,000- ¥ 30,000 | 205 (48.6) | 217 (51.4) |  |  |  |
| > ¥ 30,000 | 81 (53.6) | 70 (46.4) |  |  |  |
| Unknown | 245 (49.2) | 253 (50.8) |  |  |  |
| Chronic somatic diseases |  |  |  | 1.03 | 0.311 |
| No | 829 (50.3) | 818 (49.7) |  |  |  |
| Yes | 58 (45.7) | 69 (54.3) |  |  |  |
| Family history of mental illness |  |  |  | 2.61 | 0.106 |
| No | 881 (50.2) | 874 (49.8) |  |  |  |
| Yes | 6 (31.6) | 13 (68.4) |  |  |  |
| Anxiety and depressive symptoms |  |  |  | 0.28 | 0.599 |
| No | 492 (49.4) | 503 (50.6) |  |  |  |
| Yes | 395 (50.7) | 384 (49.3) |  |  |  |

**Supplementary Table 3.** Sensitivity analysis of risk and protective factors of change in insomnia symptoms.

|  | OR (95% CI) | |
| --- | --- | --- |
|  | New-onset vs. Resistance | Persistence vs. Remission |
| **Demographic characteristics** |  |  |
| Sex (male as Ref.) | 0.94 (0.82-1.08) | 0.94 (0.77-1.15) |
| Grade (grade 8 as Ref.) |  |  |
| Grade 5 | 1.28 (1.04-1.57)^*^ | 1.34 (1.01-1.78)**^*^** |
| Grade 6 | 1.14 (0.91-1.44) | 1.12 (0.83-1.52) |
| Grade 7 | 1.07 (0.87-1.31) | 0.99 (0.75-1.30) |
| Private school (public school as Ref.) | 1.05 (0.90-1.22) | 1.04 (0.84-1.29) |
| Boarding at School (no as Ref.) | 0.95 (0.73-1.23) | 1.13 (0.79-1.61) |
| No sibling (more than one sibling as Ref.) | 0.97 (0.82-1.16) | 0.93 (0.72-1.21) |
| Poor parental marital status (married as Ref.) | 1.20 (0.89-1.63) | 1.32 (0.91-1.91) |
| Family income (monthly) (< ¥ 12,000 as Ref.) |  |  |
| ¥ 12,000- ¥ 30,000 | 0.98 (0.83-1.16) | 1.15 (0.89-1.49) |
| > ¥ 30,000 | 1.04 (0.79-1.37) | 0.88 (0.60-1.27) |
| Unknown | 0.99 (0.82-1.19) | 1.10 (0.86-1.40) |
| Chronic somatic diseases (no as Ref.) | 1.00 (0.70-1.43) | 1.18 (0.81-1.72) |
| Family history of mental illness (no as Ref.) | 0.83 (0.46-1.49) | 1.86 (0.69-5.06) |
| Anxiety and depressive symptoms (no as Ref.) | 0.95 (0.77-1.16) | 0.86 (0.70-1.05) |
| **“Double Reduction” policy-related variables** |  |  |
| Reduced homework (no as Ref.) | **0.78 (0.70-0.86)^***^** | 0.85 (0.66-1.09) |
| More extracurricular activities (no as Ref.) | 0.90 (0.78-1.14) | 0.93 (0.72-1.21) |
| Increased physical activity (no as Ref.) | 1.01 (0.84-1.21) | 0.90 (0.70-1.15) |
| More family time (no as Ref.) | **0.62 (0.53-0.73)****^***^** | **0.54 (0.43-0.68)^***^** |
| Reduced academic pressure (no as Ref.) | **0.77 (0.66-0.88)^***^** | **0.69 (0.54-0.84)^***^** |

**Note:** ^*^ *p* < 0.05, ^**^ *p* < 0.01, ^***^ *p* < 0.001. OR, odds ratio; 95%CI, 95% confidence interval; Ref, reference.

**Supplementary Table 4.** Sex-stratified logistic regression analysis for policy-related indicators and changes in insomnia symptoms.

|  | New-onset vs. Resistance, OR (95% CI) ^a^ | |  | *p* for interaction | Persistence vs. Remission, OR (95% CI) ^a^ | |  | *p* for interaction |
| --- | --- | --- | --- | --- | --- | --- | --- | --- |
|  | Male | Female |  |  | Male | Female |  |  |
| Reduced homework |  |  |  | 0.425 |  |  |  | 0.759 |
| No | 1.00 (reference) | 1.00 (reference) |  |  | 1.00 (reference) | 1.00 (reference) |  |  |
| Yes | **0.71 (0.55-0.87)^***^** | 0.84 (0.70-0.98)^*^ |  |  | 0.81 (0.53-1.24) | 0.88 (0.64-1.20) |  |  |
| More extracurricular activities |  |  |  | 0.095 |  |  |  | 0.892 |
| No | 1.00 (reference) | 1.00 (reference) |  |  | 1.00 (reference) | 1.00 (reference) |  |  |
| Yes | 1.13 (0.85-1.50) | 0.80 (0.62-1.05) |  |  | 0.91 (0.59-1.40) | 0.93 (0.67-1.29) |  |  |
| Increased physical activity |  |  |  | 0.236 |  |  |  | 0.899 |
| No | 1.00 (reference) | 1.00 (reference) |  |  | 1.00 (reference) | 1.00 (reference) |  |  |
| Yes | 0.88 (0.67-1.16) | 1.12 (0.88-1.44) |  |  | 0.91 (0.60-1.39) | 0.89 (0.65-1.22) |  |  |
| More family time |  |  |  | 0.055 |  |  |  | 0.523 |
| No | 1.00 (reference) | 1.00 (reference) |  |  | 1.00 (reference) | 1.00 (reference) |  |  |
| Yes | **0.53 (0.42-0.67)^***^** | **0.71 (0.58-0.88)^***^** |  |  | **0.49 (0.34-0.71)^***^** | **0.57 (0.42-0.76)^**^****^*^** |  |  |
| Reduced academic pressure |  |  |  | 0.083 |  |  |  | 0.662 |
| No | 1.00 (reference) | 1.00 (reference) |  |  | 1.00 (reference) | 1.00 (reference) |  |  |
| Yes | 0.86 (0.72-1.03) | **0.66 (0.52-0.84)^***^** |  |  | 0.74 (0.51-1.09) | 0.67 (0.48-0.92)^*^ |  |  |

**Abbreviation:** OR, odds ratio; 95% CI, 95% confidence interval.

^a^ Adjusted for grade, school types, boarding at school, number of siblings, parental marital status, family income, chronic somatic diseases, family history of mental illness, and anxiety and depressive symptoms.
